# A ‘giant’ intraluminal lipoma presenting with intussusception in an adult: a case report

**DOI:** 10.1186/1752-1947-6-370

**Published:** 2012-10-29

**Authors:** Peter A Ongom, Henry Wabinga, Robert L Lukande

**Affiliations:** 1 Department of Surgery, Colorectal Surgery Unit, School of Medicine, Makerere College of Health Sciences, Makerere University, PO Box 7072, Kampala, Uganda; 2 Department of Pathology, Histopathology Unit, School of Biomedical Sciences, Makerere College of Health Sciences, Makerere University, PO Box 7072, Kampala, Uganda

**Keywords:** Colocolonic, giant polyp, hemicolectomy, intestinal obstruction, intraluminal, intussusception, lipoma, submucosal

## Abstract

**Introduction:**

Intussusception is an uncommon cause of intestinal obstruction in adults. It usually presents with typical features of intestinal obstruction, and is associated with the presence of a ‘lead point’ for the invaginated portion. This ‘lead point’ is rarely an intraluminal, submucosal lipoma.

**Case presentation:**

We describe the case of a 64-year-old African-Ugandan woman of Bantu ethnicity who presented with features of intestinal obstruction secondary to intussusception. She was treated operatively. A left colocolonic invagination was found with the interssusceptum having a giant polyp. A left hemicolectomy was performed. A histopathological examination revealed a polypoid, submucosal lipoma.

**Conclusions:**

In resource-rich countries, most cases of colonic intraluminal polyps are detected through colonoscopy during routine medical check-ups. With limited resources in our region, many tumors present as intestinal obstructions secondary to intussusception. Even then, most are associated with adenomas and malignancies. Rarely are polypoid, submucosal lipomas found. In our patient’s case a polyp of 9.5cm at its widest dimension is of particular interest. A lesson to learn is that the differential diagnosis for intussusceptions in resource-poor countries should be broadened to include submucosal lipomas.

## Introduction

Intussusception is the invagination of a segment of the intestine into the lumen of another immediately adjacent segment. This is usually in a proximal-to-distal fashion. Adult intussusception is relatively rare, constituting less than 5 percent of intussusception cases [1]. In developed countries, they have an incidence of two to three per 1,000,000 per year, representing 1 to 3 percent of all cases of intestinal obstructions [2,3]. There is a demonstrable cause in the majority of cases, usually an intraluminal neoplasm. A number of studies point to a 70 to 90 percent existence of an underlying gut pathological cause [2,3]. These are mainly polyps and colonic malignancies. In contrast, childhood intussusception is a leading cause of intestinal obstruction.

The pathophysiologic mechanism is due to peristaltic movement of a ‘lead point’ forming the apex of an intussusceptum (invagination). In turn, the commonest ‘lead points’ are colonic malignant tumors in up to 60 percent of cases [2]. Benign tumors constitute the majority of the rest of the cases. Lipomas (benign, non-epithelial tumors) are the second most frequently occurring benign tumors of the large bowel (0.2 to 26 percent) [4], the commonest being adenomas. Colonic lipomas are largely asymptomatic. Many are found incidentally at colonoscopy [5]. The median age at the time of diagnosis ranges from 50 to 69 years [2], and the sex prevalence is insignificant [6]. Involvement of multiple sites has been described [4].

This case is important because it provides us with an unexpected and unusual presentation of intussusception. We illustrate the diagnostic and surgical challenges encountered and the effort made to manage such patients in resource-limited settings. It demonstrates the importance of screening patients for early diagnosis and the complications associated with symptomatic cases [7].

## Case presentation

A 64-year-old African-Ugandan woman of Bantu ethnicity presented to Mulago National Hospital following referral from a private health facility 80 miles from Kampala, Uganda’s capital. Though the referral was to the local regional referral hospital, her relatives opted for the national referral hospital. She had experienced symptoms of abdominal pain and partial constipation for one week. The pain was characteristically colicky in nature. One day prior to admission, she developed mucoid, bloody stool motions, but had no frank diarrhea or distinctively foul-smelling stool. She was also unable to pass flatus.

She had generally been unwell for over one year, with a dull abdominal ache of insidious onset; the pain was on and off, located mainly in the left hypochondrium and lumbar region. The ache progressed to cramp-like pain, especially after meals. Associated with this was diffuse abdominal bloating and flatulence with pellet-like stool motions. Her pain was slightly relieved by passing flatus. Otherwise, she had neither had hematochezia during that time, nor had she felt any swelling in her abdomen. Her appetite was good and she felt she had neither gained nor lost weight. She had not had dyspeptic symptoms and had not been vomiting.

No history of chronic disease was reported; specifically, diabetes mellitus and hypertension. She had a total abdominal hysterectomy five years ago (at the age of 57 years) for what she described as uterine fibroids and ‘continued’ menstrual periods. However, there were no accompanying medical records to back this. She was multiparous (para 7+0), and was not aware of any familial-associated gastrointestinal, breast, or genital malignancy.

On physical examination, she was an elderly woman in pain. She was moderately dehydrated, neither jaundiced nor anemic, and afebrile. Her abdomen was grossly distended, more peripherally, though generally amorphous. There was no visible peristalsis. She had a fine, midline, sub-umbilical scar. Superficially, there was generalized, mild tenderness, though with no characteristic features of peritonitis. This was tympanitic to percussion, and bowel sounds were reduced. A digital rectal examination revealed a largely empty rectum with a normal mucosa, and no masses were felt. There were small amounts of bloody, mucoid stool. Her pulse was 82 beats per minute and blood pressure 115/80mmHg. Her chest was clear. Other systems were unremarkable.

**Figure 1:**
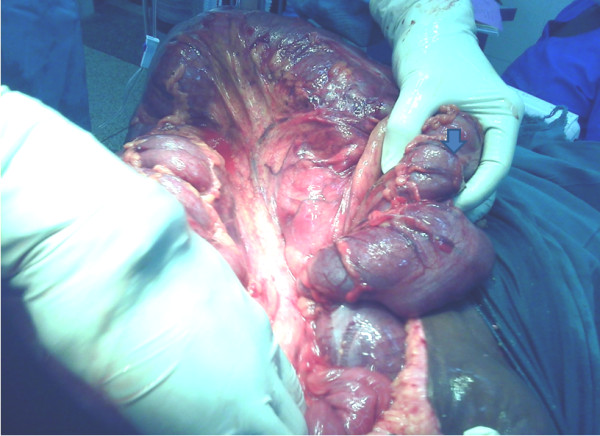
**Intraoperative photograph.** The photograph shows the surgeon’s hand partially ‘grasping’ a polypoid, intraluminal mass (arrow) in the region of the transverse colon. This followed reduction of the transverse colon’s invagination into the descending colon. The splenic ligament has been freed.

A diagnosis of large bowel intestinal obstruction secondary to intussusception was made. Our patient was resuscitated and prepared for an exploratory laparotomy. This was performed through a midline incision. On opening the peritoneum, bloodstained, odorless fluid exuded, and grossly distended loops of bowel were seen. These were delivered and inspected. A colocolonic intussusception was identified with the invagination extending from the middle part of the transverse colon to the descending colon, looping through the splenic flexure. No intraperitoneal adhesions were found. The gut involved was moderately edematous, though neither friable nor gangrenous. The intussusceptum was gently ‘milked’ proximally and freed. A large, polypoid, intraluminal mass was palpated (Figure 1). It had a small stalk attached to the anti-mesenteric border of the distal third of the transverse colon. Notably, she also had only 6cm to 8cm of retroperitoneal descending colon, the rest being intraperitoneal. A left hemicolectomy was performed. Fresh, fully viable and non-edematous resection ends were fashioned. This allowed us to perform a primary, end-to-end anastomosis of the mid-transverse colon to the rectum, in two layers. The anterior abdominal wall was closed in layers.

**Figure 2:**
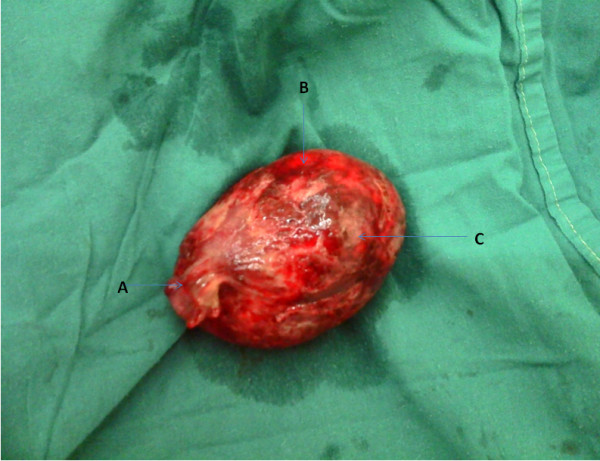
**Photograph showing the lipomatous polyp.** This photograph shows the following labeled structures: (**A**) stalk, (**B**) hemorrhagic areas, (**C**) necrotic mucosal areas. Shown is a soft, hemorrhagic, inflamed, and pedunculated polypoid lipoma. It was detached from the transverse colon lumen, into which it ‘dangled’. Actual size (immediately post-operatively) was 9.5×7.2×6.0cm.

The resected portion of gut revealed an intraluminal polyp as the ‘lead point’ for the intussusceptum. Its stalk (1.5cm) was attached approximately 12cm proximal to the splenic flexure. It measured 9.5×7.2×6.0cm, and was uniformly soft. The mucosa had patches of hyperemia, edema and hemorrhage (Figure 2).

She generally had a good post-operative recovery. Post-operative laboratory investigation results were unremarkable. She was discharged on the 10th post-operative day. Her subsequent follow-up visits showed steady progress, with a return to normal diet and gradual normalization of bowel motions and stool consistency. We continue to review her monthly, and intend to perform a barium enema and colonoscopy to rule out any other lipomas and other pathological lesions.

**Figure 3:**
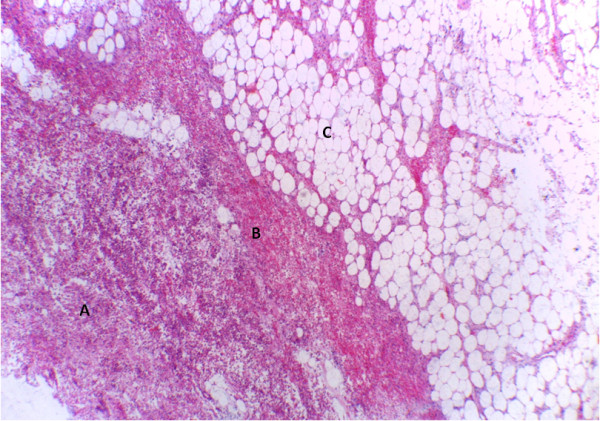
**Photomicrograph of mucosal and submucosal areas.** Hematoxylin and eosin stained section of gut showing necrotic and inflamed mucosa (**A**). The submucosa contains areas of hemorrhage (**B**) and mature adipocytes (**C**) (magnification ×40, objective lens).

Pathological examination revealed a polypoid mass, macroscopically fatty and nodular in nature. It measured 9cm at its widest diameter (Figure 2). Histologically, there was non-specific, acute inflammation within the lobules of fat seen (Figure 3). It was concluded that this was an infected submucosal lipoma.

## Discussion

Colonic lipomas, in life, are mostly asymptomatic. Going by autopsy prevalence reports, the vast majority are incidental findings (0.2 to 0.3 percent). Our patient’s case described here manifested with relatively typical symptoms of an intraluminal, lipomatous polyp causing intussusception.

**Figure 4:**
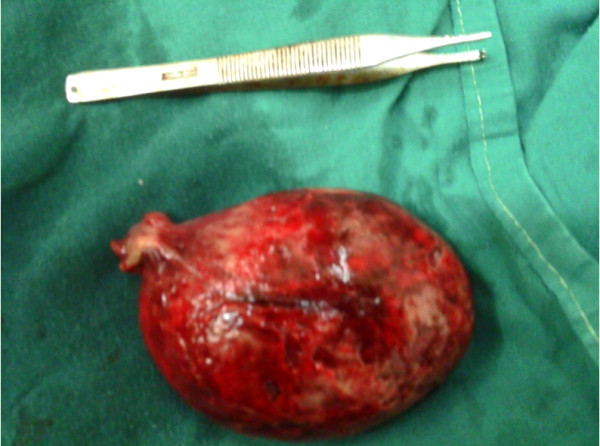
**Photograph showing relative size of the lipoma.** Demonstration of the relative size of the lipoma. Lying alongside it is a dissecting forceps, for the purpose of comparison.

The size of the lipoma has a bearing in predicting its symptoms, presentation and management. Generally, those less than 2cm (widest dimension) tend to be asymptomatic, and may be electively removed endoscopically (colonoscopic resection) [4,8]; those greater than 2cm are usually managed by surgical resection [9-11]. Approximately 75 percent of lipomas exceeding 2cm at the widest diameter have a presentation similar to that of our patient’s case. An ulcerated lead point for the intussusceptum causes bleeding in up to 7 percent of patients with large lipomas [12] (Figure 4).

Ideally, management should start with screening around the peak age of incidence in the fifth and sixth decades of life. This involves abdominal and rectal examination, barium enema and angiography, followed by colonoscopy. Biopsy through colonoscopy can be both diagnostic and curative, and remains the definitive diagnostic investigation of choice.

There are two broad treatment options; conservative management (reduction with barium or air [13], and close observation) and surgical intervention. Conservative treatment is used for those in the younger age group and patients with no demonstrable anatomical ‘lead point’, following radiological investigations. Patients with special medical conditions such as hemophilia [14] and ulcerative colitis [15] are resuscitated, observed and given medicinal treatment for their respective conditions. The use of barium and air is avoided.

Surgical intervention has two options: colonoscopic snaring and resection, and open surgery. The treatment of choice, in centers that have it available, is colonoscopic resection. Its use is usually restricted to small and medium sized polyps (<7cm at widest diameter). Larger ones (>7cm) are often treated with open surgery [11]. It is also used in the event of strangulation and malignancy. In resource-limited settings such as Uganda, open surgery is still the first choice of intervention regardless of polyp size.

The open surgical procedure recommended for cases of intussusception in adults (middle aged and older) is wide surgical resection as opposed to manipulative reduction [12]. This reduces the risk of ‘manual’ tumor spread. The basis of this caution is the higher frequency of colonic intussusceptions associated with malignancy [2]. However, this should be assessed on a case-by-case basis.

Colocolonic intussusception with a giant submucosal lipoma represents a rare manifestation of a benign, colonic polyp. It is challenging in resource-limited settings. The extent of resection depends on tumor size, length of intussusceptum/intussuscipiens, and the amount of inflammation. An ‘intussusception maneuver’ may be performed first, followed by a hemicolectomy. In our case the same was performed in order to evaluate the bowel better. It is useful to investigate for any other polyps (not only lipomatous) as part of the patient’s follow-up plan. Usually, there is only a solitary lesion, but multiple localizations are important to identify.

Rare causes of colonic intestinal obstruction have to be taken into account, since they mimic the features of the case in point. There are a number of them, most recorded in the literature as case reports. It is therefore necessary to rule out the following specific conditions: colonic mucosubmucosal elongated polyp, inflammatory myoglandular polyp (Nakamura polyp), inflammatory fibroid polyp, gastrointestinal stromal tumor (GIST), liposarcoma of the colon, and giant pseudopolyp with ulcerative colitis [15]. All these conditions can be readily identified using routine histopathological techniques.

## Conclusions

Colonic lipomas, with their unusual properties, present difficulties in pre-operative differentiation between malignant and benign colonic neoplasms. Furthermore, the investigative procedures are challenging; this is particularly the case in resource-limited countries.

This case offers a valuable lesson. We had a patient in whom a ‘giant’, intraluminal lipoma (submucosal type) was identified at the time of exploratory laparotomy for large bowel obstruction, secondary to intussusception. Our practice is based on anecdotal evidence, which usually focuses our differential diagnosis on an adenocarcinoma or adenoma as the cause. In our institution, this is the first documented case of this type. There is therefore a need to include lipomas on our list of definite causes of intussusception in adults.

Screening for colonic pathology starting from the onset of middle age is a useful strategy. This enables earlier detection of this condition among patients with suspicious symptomatology. This lessens the burden of emergency surgery and prolonged hospital stay. Rectal examination, occult blood testing, barium studies, flexible colonoscopy and computed tomography (CT) are all options for screening and/or diagnosis.

For our patient’s follow-up, we may start with barium studies (enema). This will be proceeded by flexible colonoscopy (barium studies may be excluded). In Uganda, colonoscopy has been available for over 15 years, though it is expensive. Our patient comes from a middle-class family, which by Ugandan standards means colonoscopy is affordable. Any other colonic lesions can also be identified, including multiplicity of lipomas. CT scans are useful for subserosal types and inconclusive findings with other methods.

## Consent

Written, informed consent was obtained from the patient for publication of this case report and any accompanying images. A copy of the written consent is available for review by the Editor-in-Chief of this journal.

## Competing interests

The authors declare that they have no competing interests.

## Authors’ contributions

OAP managed our patient peri-operatively, performed the operation, and wrote the manuscript. WH prepared, examined and analyzed the specimen and histopathological slides. LRL examined and described the histopathological slides. WH and LRL edited the manuscript. All authors read and approved the final manuscript.
